# The Effects of Sesquiterpene Lactones on the Differentiation of Human or Animal Cells Cultured *In-Vitro*: A Critical Systematic Review

**DOI:** 10.3389/fphar.2022.862446

**Published:** 2022-04-04

**Authors:** Sepand Tehrani Fateh, Sahand Tehrani Fateh, Faezeh Shekari, Majid Mahdavi, Amir Reza Aref, Amir Salehi-Najafabadi

**Affiliations:** ^1^ School of Medicine, Shahid Beheshti University of Medical Sciences, Tehran, Iran; ^2^ School of Medicine, Tehran University of Medical Sciences, Tehran, Iran; ^3^ Department of Stem Cells and Developmental Biology, Cell Science Research Center, Royan Institute for Stem Cell Biology and Technology, ACECR, Tehran, Iran; ^4^ Department of Biology, Faculty of Natural Science, University of Tabriz, Tabriz, Iran; ^5^ Belfer Center for Applied Cancer Science, Department of Medical Oncology, Dana-Farber Cancer Institute, Harvard Medical School, Boston, MA, United States; ^6^ Translational Sciences, Xsphera Biosciences Inc., Boston, MA, United States; ^7^ Department of Microbiology, School of Biology, University College of Science, University of Tehran, Tehran, Iran; ^8^ Research Center for New Technologies in Life Science Engineering, University of Tehran, Tehran, Iran

**Keywords:** sesquiterpene lactone, lactone, differentiation, signaling pathways, cancer, negative results, *in-vitro*, systematic review

## Abstract

Cellular differentiation is pivotal in health and disease. Interfering with the process of differentiation, such as inhibiting the differentiation of adipocytes and inducing the differentiation of cancer cells, is considered a therapeutic approach. Sesquiterpene lactones, primarily found in plants, have been attracted attention as differentiating/dedifferentiating agents tested on various human or animal cells. However, a consensus on sesquiterpene lactones’ effects and their mechanism of action is required. In this sense, through a systematic review, we have investigated the differentiating/dedifferentiating effects of sesquiterpene lactones on human or animal cells. 13 different cell lines originated from humans, mice, and rats, in addition to the effects of a total of 21 sesquiterpene lactones, were evaluated in the included studies. These components had either inducing, inhibiting, or no effect on the cells, mediating their effects through JAK-STAT, PI3K-Akt, mitogen-activated protein kinases, NFκB, PPARγ pathways. Although nearly all inducing and inhibiting effects were attributed to cancerous and normal cells, respectively, this is likely a result of a biased study design. Few studies reported negative results along with others, and no study was found reporting only negative results. As a result, not only are the effects and mechanism of action of sesquiterpene lactones not vivid but our knowledge and decisions are also misconducted. Moreover, there is a significant knowledge gap regarding the type of evaluated cells, other sesquiterpene lactones, and the involved signaling pathways. In conclusion, sesquiterpene lactones possess significant effects on differentiation status, leading to potentially efficient therapy of obesity, osteoporosis, and cancer. However, reporting negative results and further investigations on other cells, sesquiterpene lactones, and signaling pathways are highly suggested to pave the path of sesquiterpene lactones to the clinic more consciously.

## Introduction

Cellular differentiation is perhaps one of the most crucial biological processes in almost all living creatures. Cells reorganize their facilities and redirect their functions to adapt to certain needs through differentiation; however, not all these reorganizations and redirections are commonly considered differentiation (Márquez-Zacarías et al., 2021). Changes of preadipocytes to adipocytes, hematopoietic stem cells to erythrocytes, macrophages to osteoclasts, and stem cells to epithelial cells are some instances of cellular differentiation.

Various human diseases are at least partially related to the process of differentiation. The early reports on the involvement of the differentiation process in cancer formation can be traced back to at least 100 years ago (Mallory, 1910). Over the years, the components of the differentiation process were identified, and differentiation therapy of cancer emerged (de Thé, 2018). In contrast, inhibition of differentiation is required to achieve therapeutic goals in other diseases. For instance, suppressing the differentiation of preadipocytes to adipocytes or macrophages to osteoclasts would prevent metabolic disorders (Gu et al., 2018) or osteoporosis (Jing et al., 2018), respectively.

They are currently medications such as all-trans retinoic acid (or known as Tretinoin, see (Wang and Chen, 2008) for clinical trials) for Acute Promyelocytic Leukemia treatment (Warrell et al., 1991) and statins (e.g., Atorvastatin (McCrindle et al., 2003)) for hyperlipidemia treatment (Nakata et al., 2006) that exert their therapeutic function through differentiation. There is a trend toward identifying more compounds for this purpose. Sesquiterpene lactones have been investigated extensively as differentiation-inducing/-inhibiting agents, either *in vivo* or *in vitro*. Sesquiterpene lactones can be isolated from plant extracts (Laid et al., 2008); however, semi-synthetic (Perri et al., 2019) and synthetic (Wedge et al., 2000) compounds also exist. Despite various derivations, the lactone ring and C15-terpenoids, built from three isoprene units (i.e., sesquiterpene), are shared in all members of this group (Aliarab et al., 2018). Differentiation and dedifferentiation therapies are a growing field, and sesquiterpene lactones can alter differentiation. Therefore, a comprehensive understanding of how different sesquiterpene lactones affect cell differentiation is necessary.

Two systematic reviews have investigated the effects of sesquiterpene lactones on cancer cells and skin cells (da Silva Castro et al., 2018; da Silva et al., 2020); however, no systematic review has been conducted focusing on the effect of sesquiterpene lactones on cellular differentiation, to the best of our knowledge. Herein, we have conducted a systematic review on the differentiating/dedifferentiating effects of sesquiterpene lactones on various human or animal cells, evaluated *in vitro*. Both inducing and inhibiting effects of sesquiterpene lactones on differentiation are covered in this survey to scheme a general idea regarding their effects. Moreover, this review briefly discusses the underlying mechanisms of sesquiterpene lactones’ effects on differentiation. The gap in our current knowledge about this field is identified and suggestions for future researches are also discussed. It is worth noting, due to data heterogeneity (i.e., variation in cell and lactone type, and variation in measurement tools), and lack of quantitative measurements in some studies, we were not able to conduct a meta-analysis (as in (da Silva Castro et al., 2018; da Silva et al., 2020)).

## Methods

### Search Strategy and Databases

This systematic review was conducted according to the PRISMA (Preferred Recording Items for Systematic Reviews and Meta-analyses) statement (www.prisma-statement.org). We have asked how sesquiterpene lactones would affect the differentiation status of human or animal cells in an *in vitro* experiment. A preliminary search was conducted to obtain keywords for a systematic search. “Sesquiterpene lactone” and “differentiation” were selected as primary keywords; however, the search string was formed based on other derivations of these two keywords, according to MeSH (www.ncbi.nlm.nih.gov/mesh). Scopus, Web of Science, and MEDLINE were searched for the current survey. No limitations were set in terms of date, but the language of the results was limited to English. The detailed search string for each database can be found in supplementary material 1.

### Eligibility Criteria

Any *in vitro* study on animal or human cells (population) which the treatment was purified sesquiterpene lactones and not crude plant extract (exposure) with an untreated or vehicle control group, with or without a positive control group (comparison), assessing the status of cellular differentiation, either induction or inhibition (outcome) were included. The article should clearly state the occurrence of differentiation with any manifestation as the primary outcome (e.g., morphological, biochemical, gene expression profile alternation, and biomarkers expression). Any interpretation by the authors of the current systematic review was prohibited. Non-comparative studies, including abstracts, opinions, reviews, and systematic reviews, were excluded. The studies evaluating the concurrent (synergistic or antagonistic) effect of sesquiterpene lactones and another component on the induction of differentiation were excluded. The studies reporting the duration and concentration of treatment as a range were excluded since the exact condition of induction or inhibition of differentiation and a general conclusion could not be obtained from these studies. All cells with either cancerous or non-cancerous (at any stage of differentiation) were acceptable. In addition, genetic modification of the cells was not acceptable since it imposes an unknown impact on the cellular physiology and the effects sesquiterpene lactones. Moreover, through the last step of screening, the studies were screened in terms of significant inaccuracy in study design and analysis with major impact on the interpretation of results and those with no statistical analysis in the presentation and explanation of results, not specified duration and concentration of treatment, and not dedicated control group.

### Study Selection

The extracted studies from the databases were first screened for the key components of the question of the review (population, exposure, comparison, and outcome) by their title and abstract by two authors independently. Similarly, the included studies from the last stage were assessed by their full text by two authors independently. Discrepancies were resolved by discussion or a third reviewer if they remained.

### Data Extraction

The extraction of data was conducted by two authors independently. Discrepancies were resolved by discussion or a third reviewer if they remained. The following data were extracted: the name of publication, cell name and type, the origin of the cell (species), Kind of the cell (i.e., cancer cell, differentiated cell, and stem cell), sesquiterpene lactone name, the origin of sesquiterpene lactone (i.e., natural, semi-synthetic, synthetic), the concentration of treatment, duration of treatment, differentiation status after treatment (i.e., induction, inhibition, or no response), control group, detection method, involved genes, and pathways.

### Quality Assessment

Since no standard checklist exists for *in vitro* studies, we have created our criteria based on the most important components of *in vitro* studies to adapt our requirements. We have assessed studies by nine items. The items are designed to evaluate the selected studies after the study selection process regarding their precise, rigor, and comprehensive description of study design and data presentation. No study, selected according to the eligibility criteria will be eliminated based on the quality assessment. The items were answered by yes or no. Quality assessment was done independently by two authors. Discrepancies were resolved by discussion or a third reviewer if they remained. The traffic light plot is created using robvis tool (McGuinness and Higgins, 2020).

### Data Synthesis

The studied cells were collected, categorized into cancer, normal, and stem cells, and their developmental origin was determined. The effects of sesquiterpene lactones on differentiation status regarding the dose range and duration of treatment were investigated. We further evaluated if some sesquiterpene lactones had double responses in single or multiple cell lines. The involved pathways were determined in inhibiting and inducing effect groups and compared to obtain a general scheme of the underlying mechanism in each group. The biological assays used in the studies were collected and analyzed to demonstrate the level of evidence on the status of differentiation.

### Protocol Registration

The protocol of this systematic review was registered on 28 September 2021, on Open Science Framework (http://www.osf.io) before the stage of data extraction with a registration DOI of 10.17605/OSF.IO/T7QPJ.

## Results and Discussion

### Selected Studies

**FIGURE 1:**
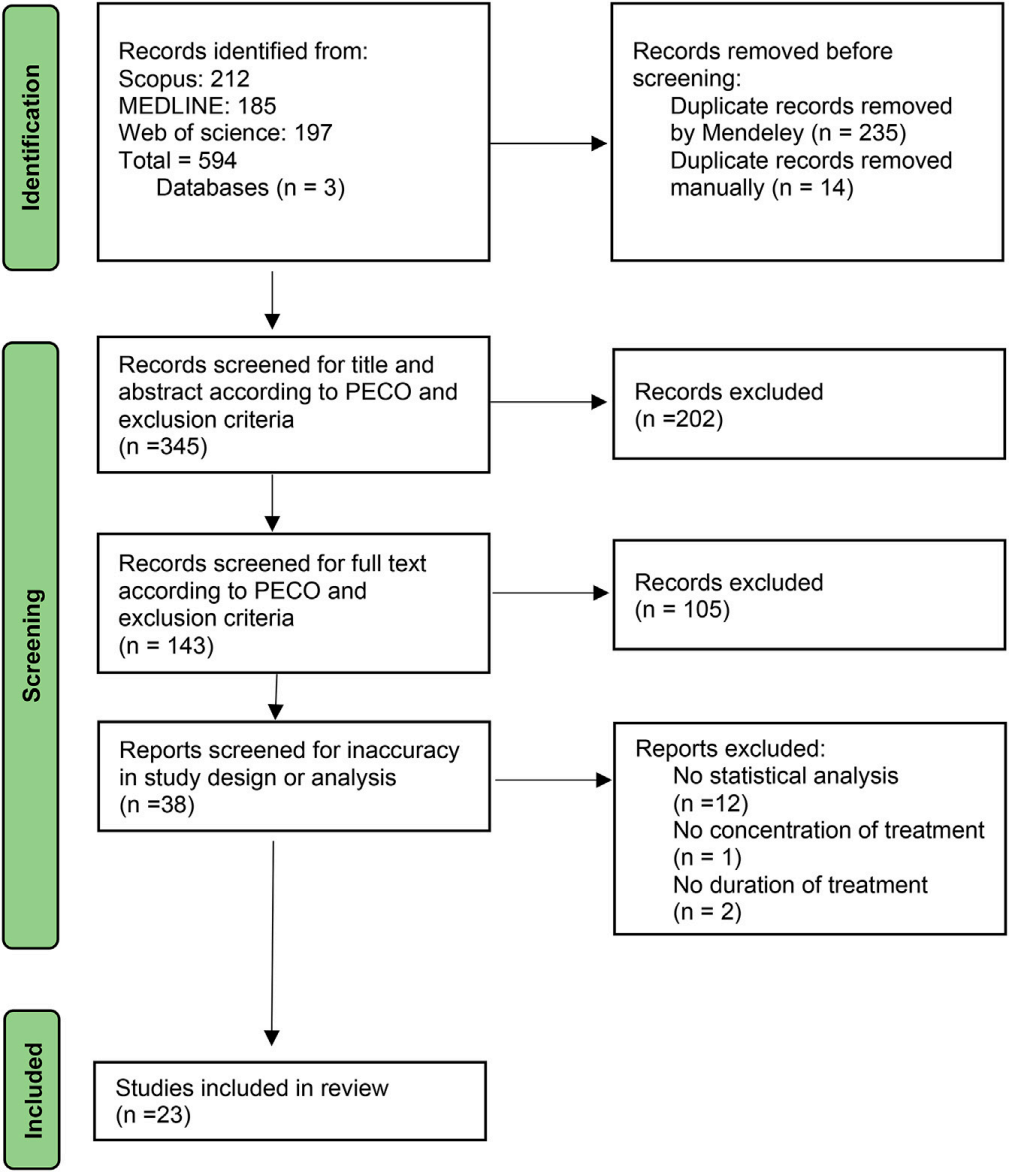
Flow chart of study inclusion and exclusion steps and study selection. PECO: Population, Exposure, Comparison, Outcome

A total of 594 studies were identified from Scopus, MEDLINE, and Web of Science databases regarding the keywords. After eliminating duplicates manually or by Mendeley, 345 studies remained for the title- and abstract-screening. 202 and 105 studies failed to fulfill the inclusion criteria in the title and abstract and full-text screening, respectively. The remaining 38 studies screened for inaccuracy in study design or statistical analysis. Despite meeting the inclusion criteria, 12 studies lacked statistical analysis, 1 study stated no treatment concentration, and two studies did not report the duration of treatment accurately and therefore eliminated. As a result, 23 studies were included in the review. The follow chart of study inclusion and exclusion is demonstrated in Figure 1.

### Quality Assessment

**FIGURE 2:**
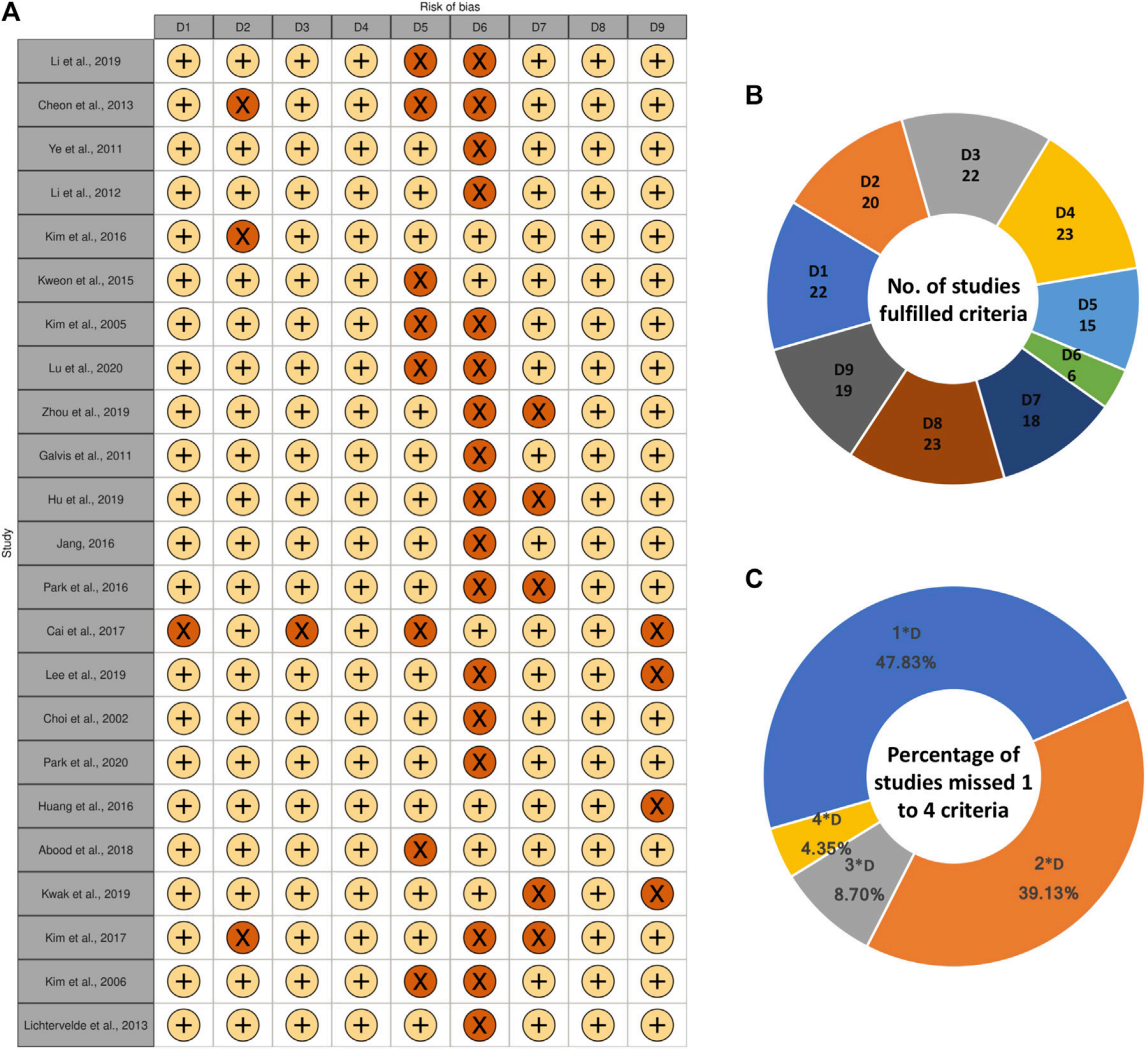
**(A)** Quality assessment of included studies illustrated in traffic light plot. D1: Does the study report a well-defined control group? D2: Can the origin (species and the cell bank obtained from) of the studied cells be confirmed? D3: Does the study mention the duration of treatment clear and accurate (Ranges are not accepted)? D4: Does the study mention the concentration of treatment clear and accurate (Ranges are not accepted)? D5: Does the study mention the condition of cell culture in details (culture medium, additives, atmosphere, temperature)? D6: Does the study mention all data accurately according to the study design? D7: Is the lactone either characterized by authors or bought from identified company? D8: Does the study mention the detection method of differentiation? D9: Does the study mention experimental repetitions? +: YES, ×: NO, **(B)** Number of studies fulfilled each criterion, **(C)** Percentage of studies which missed 1, 2, 3, or four criteria among nine criteria.

The quality assessment of included studies is illustrated in the traffic light plot (Figure 2). All studies did not fulfill at least one criteria. Among 23 studies, 17 studies failed to mention all data accurately according to the study design. About 47 and 39% of studies did not fulfill one or two criteria. Moreover, two studies did not fulfill three criteria, and one study did not fulfill four criteria. No studies were removed based on quality assessment for further steps. The studies that fail to meet these criteria are difficult to replicate, interpret, and be used in further studies. Therefore, more strict and rigor guidelines are needed to standardize the study design and results description in the articles.

### Evaluated Cells

**FIGURE 3:**
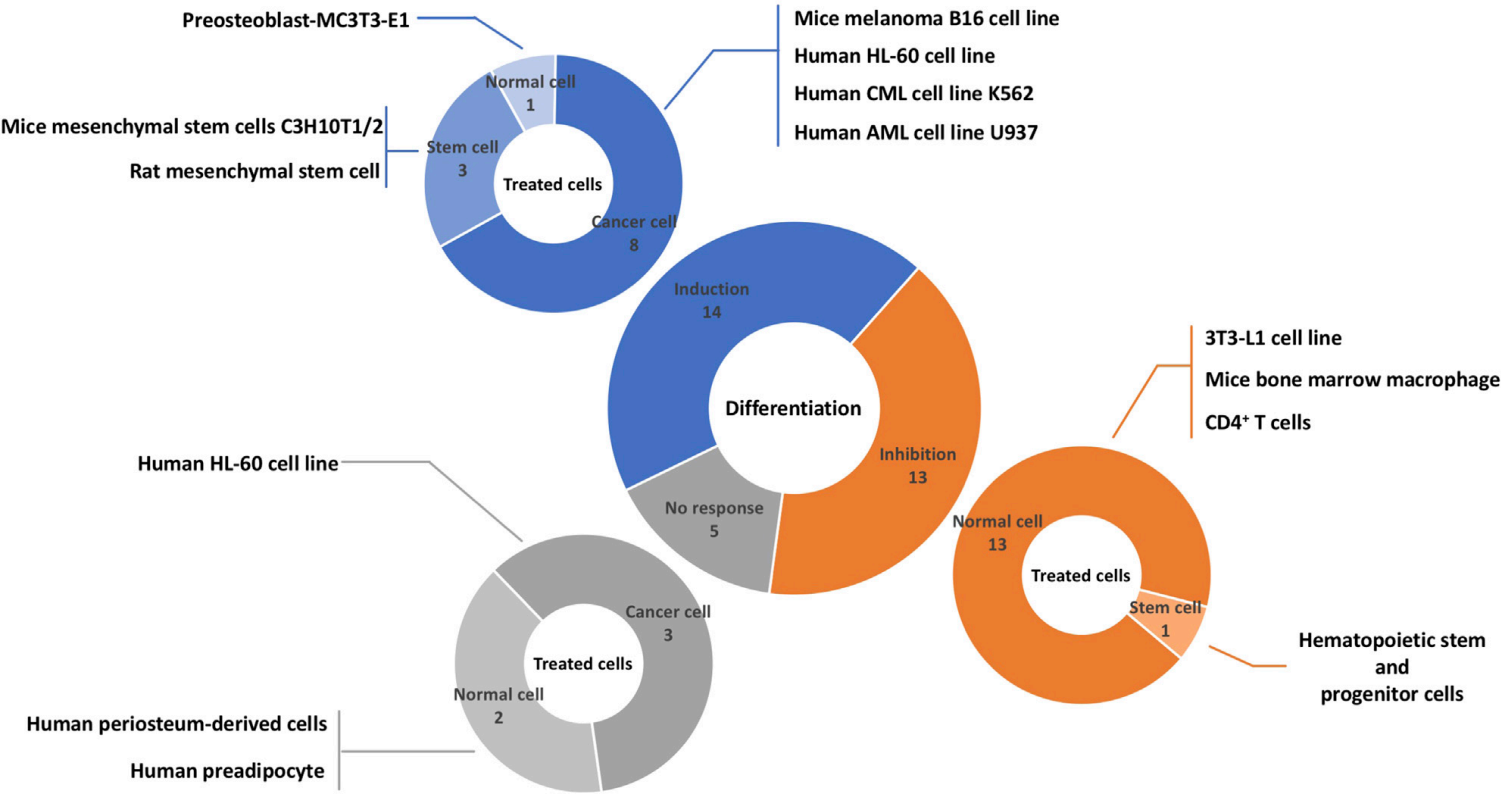
Overview of literature on the effects of sesquiterpene lactones on differentiation and affected cells. Sesquiterpene lactones either induced differentiation, inhibit it, or had no effect. The effects of sesquiterpene lactones were evaluated in normal and cancer cell lines and stem cells. The numbers represent the experiments and not papers.

A total of 13 different cell lines originated from humans, mice, and rats were evaluated in the studies (Figure 3). Among them, four cells were cancer cell lines including, mice melanoma B16 cells (Ye et al., 2011), human HL-60 cell line (Choi et al., 2002; Kim et al., 2005; Kim et al., 2006; Kweon et al., 2015), human CML cell line K562 (Huang et al., 2016; Cai et al., 2017), human AML cell line U937 (Huang et al., 2016), three cells were stem cells including rat mesenchymal stem cell (Li et al., 2012), mice mesenchymal stem cells C3H10T1/2 (Kim et al., 2017), hematopoietic stem and progenitor cells (HSPCs) (de Lichtervelde et al., 2013), and six cells were normal cell lines including, 3T3-L1 cell line (Galvis et al., 2011; Jang, 2016; Kim et al., 2016; Abood et al., 2018; Kwak and Kim, 2019), mice bone marrow macrophage (Cheon et al., 2014; Hu et al., 2019; Lee et al., 2019; Li et al., 2019; Zhou et al., 2019; Lu et al., 2020), CD4^+^ T cell (Park et al., 2016), human periosteum-derived cells (Park et al., 2020), human preadipocytes (Jang, 2016), mice MC3T3-E1 cell line (Kim et al., 2017). All cell lines except the murine melanoma B16 cell line had a mesodermal origin.

Differentiation of germ layer to endoderm, mesoderm, and ectoderm and differentiation of these lineages to specific cells and tissues involve specific signaling pathways leading to different cellular characteristics (Murry and Keller, 2008; Xie et al., 2013). Therefore, the developmental origin of cells might be influential on the quality of response to differentiating or dedifferentiating agents. There is currently a significant gap of knowledge in the effects of sesquiterpene lactones on other cells with mesodermal origins in addition to the cells with endodermal and ectodermal origins. This is of importance in both basic and clinical research. For instance, dedifferentiation is considered a crucial step in state-of-the-art therapeutic approaches, specifically regenerative medicine (Jopling et al., 2011; Tata et al., 2013). Dedifferentiation of epithelial cells or cardiomyocytes to regenerate the kidney and heart in acute renal failure and heart failure is demonstrated as an effective treatment (Jopling et al., 2010; Allison, 2020). Moreover, differentiation therapy is considered an effective treatment in various cancers (Sell, 2004). Differentiation therapy would also benefit diabetes mellitus, routing potentially from the dedifferentiation of pancreatic beta-cells (Dor and Glaser, 2013). Sesquiterpene lactones might also be found helpful in such applications, and further researches is recommended.

Investigating the effects of sesquiterpene lactones on other cells with different origins may facilitate establishing a general scheme on the mechanism of action of sesquiterpene lactones and their targets. Moreover, the general administration of sesquiterpene lactones to the body may interfere with the differentiation of unwanted targets; hence, expanding the investigations in this field to other cells would increase the ability to predict adverse effects. The effect of sesquiterpene lactones on tissues or multiple physiologically-related cells is also missed in the literature. Sesquiterpene lactones can be metabolized by certain cells, leading to active metabolites potentially interfering with the differentiation status of other cells (Yu et al., 2021).

### Sesquiterpene Lactones and Their Effects

Sesquiterpene lactones are a diverse family of bioactive molecules primarily discovered in plants. Sesquiterpene lactones play important roles in plants and are beneficial due to their anti-herbivory effects, anti-microbial activity, and allelopathic function. They may also act as a defense against ozone damage in plants (Chadwick et al., 2013). The health benefits of sesquiterpene lactones for people have been widely investigated. Their diverse bioactivities such as anti-microbial activity, anti-malaria activity, anti-tumor activity, and anti-inflammatory activity have drawn a considerable interest into their therapeutic potentials (Shoaib et al., 2017).

**FIGURE 4:**
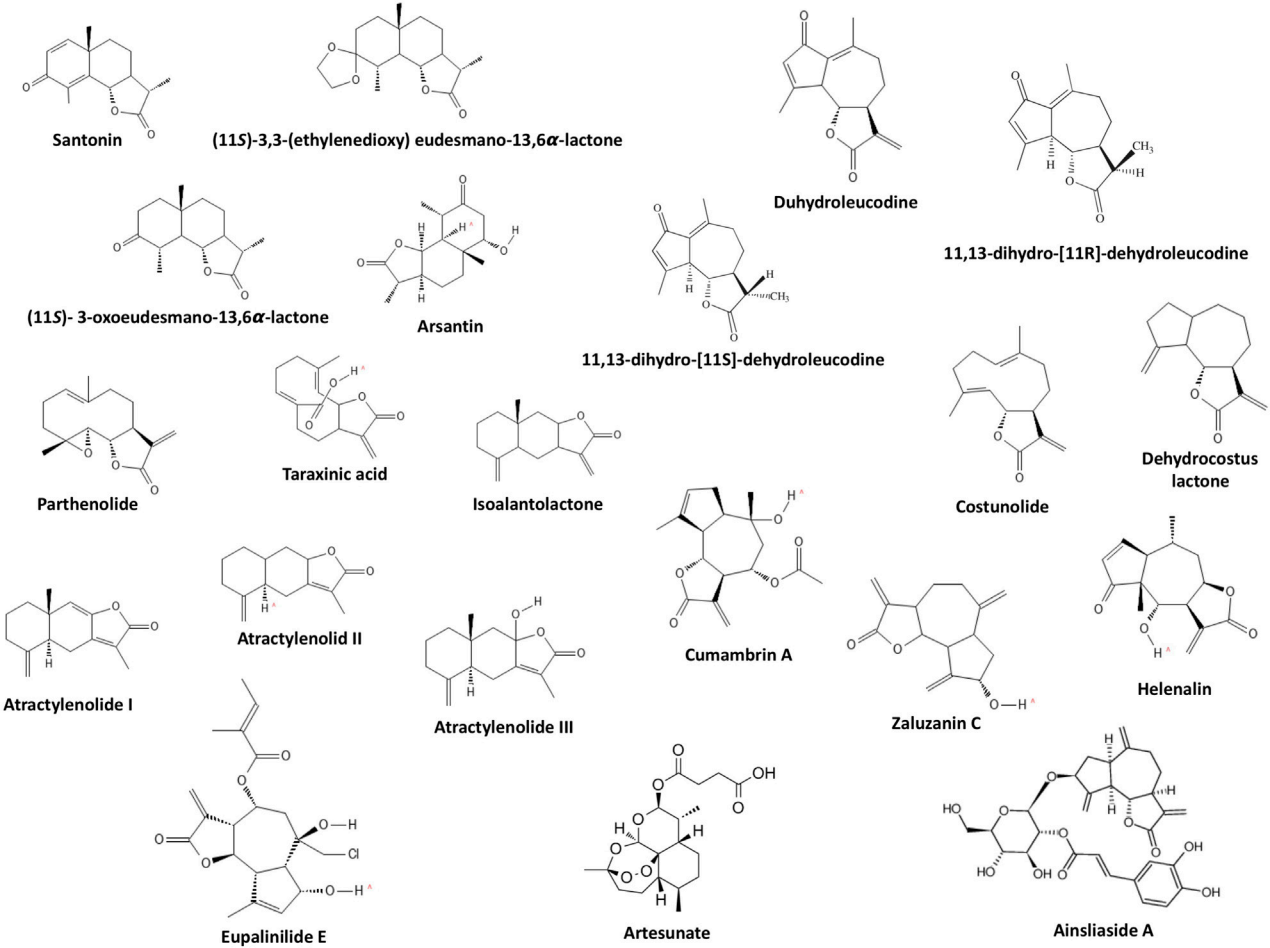
Chemical structure of evaluated sesquiterpene lactones.

A total of 21 sesquiterpene lactones were evaluated in the selected studies (Figure 4). Sixteen sesquiterpene lactones had a natural origin, and four of them were semi-synthetic (i.e., synthesized from a natural sesquiterpene lactone with further modifications). The sesquiterpene lactones had inducing, inhibiting, or no effects on the differentiation status of the cells. Through investigating the inducing effects, the cells were solely treated with sesquiterpene lactones. On the other hand, for investigating the inhibiting effects, the cells were primarily treated with approved differentiating agents in addition to sesquiterpene lactones. Three sesquiterpene lactones, including artesunate, dehydrocostus lactone, and zaluzanin C, had multiple responses in different cells lines. artesunate inhibited the differentiation of the 3T3-L1 cell line and had no effect on human preadipocytes (Jang, 2016). dehydrocostus lactone inhibited the differentiation of bone marrow-derived macrophages (also referred to as primary murine bone monocyte/macrophage precursors) (Hu et al., 2019; Lee et al., 2019; Li et al., 2019) and induced the differentiation of the K562 CML cell line (Cai et al., 2017). zaluzanin C inhibited the differentiation of the 3T3-L1 cell line (Kwak and Kim, 2019) and induced the differentiation of mesenchymal stem cells C3H10T1/2 and preosteoblast MC3T3-E1 (Kim et al., 2017). The effective dose of sesquiterpene lactones evaluated in studies ranged from 0.5 to 120 μM for inhibiting effects and 2 to 217 μM for inducing effects. The effective duration of treatment evaluated in studies ranged from 2 to 9 days for inhibiting effects and 2–7 days for inducing effects.

Sesquiterpene lactones also impose other effects on human and animal cells. Sesquiterpene lactones interfere with the cell cycle, inhibit proliferation, and induce apoptosis (Guzman et al., 2005; Yami et al., 2020). In some studies, the effective concentration to exert these effects is overlapped with the concentration effective for inducing or inhibiting differentiation [for instance as in (Guzman et al., 2005) and (Li et al., 2020)], which needs to be clarified in future studies. Moreover, the number of studies on each sesquiterpene lactone is extremely low, limiting a robust conclusion on the true effects and mechanism of actions of each molecule. More than thousands of sesquiterpene lactones exist (Acevedo et al., 2018). Investigations on these molecules are also suggested as minor structural variations between molecules would make some of them more effective for certain purposes (Schmidt, 2006; Tang et al., 2018). For instance, addition of morpholino and dimethylamino groups to sesquiterpene lactones would increase the *in vitro* activity over native sesquiterpene lactones (Zimmermann et al., 2014). In addition, extensive research on other sesquiterpene lactones would lead to a structure-response library suitable for modeling and prediction.

### Pathways Aaffected by Ssesquiterpene Lactones

**FIGURE 5:**
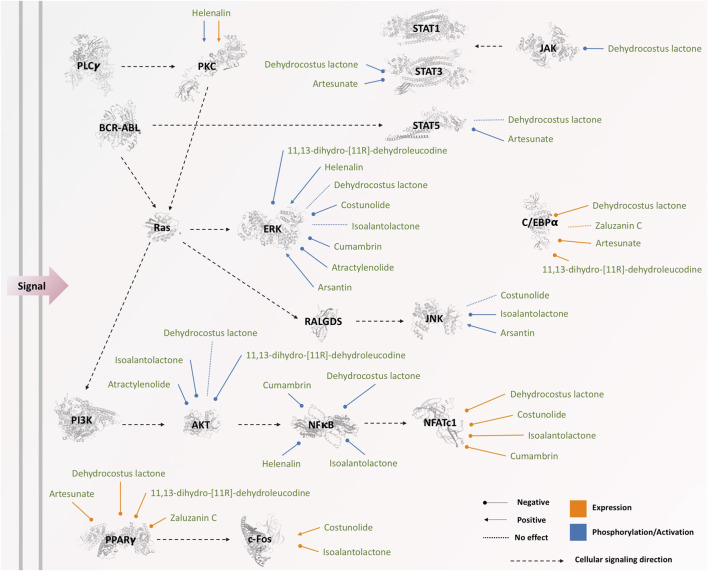
The effects of sesquiterpene lactones on different components of signaling pathways. The phosphorylation status of JAK, BCR-ABL, ERK, PKC, STATs, PI3K, AKT, and NFκB was affected by sesquiterpene lactones, while the expression of NFATc1, PKC, c-Fos, PPARγ, and C/EBPα was changed as a result of the treatment. The three-dimensional illustrations of proteins were retrieved from Protein Data Bank in Europe (PDBe) hosted by EMBL-EBI (https://www.ebi.ac.uk/pdbe).

Four main pathways, including JAK-STAT, PI3K-Akt, MAPK, and NFκB, were commonly involved in both inhibiting and inducing effects of sesquiterpene lactones, which the main components are illustrated in Figure 5. PPARγ pathway was only reported in the studies on inhibiting effects of sesquiterpene lactones; however, it was not evaluated in the studies investigating the inducing effects of sesquiterpene lactones, and its role cannot be ruled out.

### MAPK Pathway

The mammalian mitogen-activated protein kinases (MAPK) family consists of three members, including extracellular signal-reduced kinase (ERK), p38, and c-Jun NH2-terminal kinase (JNK) each exists in several isoforms (Kim and Choi, 2010). In order to activate a MAPK, MAPK-kinase kinase should phosphorylate MAPK kinase, which in turn the latter will phosphorylate and activate MAPK (Wagner and Nebreda, 2009). Activated MAPK plays a pivotal role in cell proliferation, apoptosis, inflammation, and differentiation (Wagner and Nebreda, 2009).

The phosphorylation of some components from the MAPK pathway, such as ERK, p38, and JNK, was evaluated during inducing or inhibiting differentiation via sesquiterpene lactones in some of the studies (*n* = 10) included in this systematic review (Table 1). The level of phosphorylation of these components was either increased (Kim et al., 2005; Kweon et al., 2015), decreased (Ye et al., 2011; Park et al., 2016; Abood et al., 2018; Zhou et al., 2019; Lu et al., 2020), or unchanged (Galvis et al., 2011; Cheon et al., 2014; Park et al., 2016; Li et al., 2019; Lu et al., 2020); however, there was no association between the status of phosphorylation and induction or inhibition of differentiation. The expression of c-Fos downstream of the MAPK pathway is also affected by sesquiterpene lactones (Cheon et al., 2014; Lu et al., 2020). Two studies (Galvis et al., 2011; Li et al., 2019) reporting inhibitory effects of dehydrocostus lactone on differentiation unanimously stated that dehydrocostus lactone does not affect the phosphorylation of ERK, and two studies (Cheon et al., 2014; Park et al., 2016) reporting inhibitory effects of costunolide unanimously stated that costunolide does not affect the phosphorylation of JNK. Other studies did not report opposite observations; however, further investigation is required to confirm the effects of dehydrocostus lactone and costunolide on differentiation since the current studies are insufficient to obtain a general conclusion.

**TABLE 1:** Sesquiterpene lactones and their effects.

Lactone	Treated Cell	Status of Differentiation	Effective Dosage range	Effective Duration range	Affected pathway	References
11,13-dihydro-[11R]-dehydroleucodine	3T3-L1	Inhibition	80 µM	9 days	- (a), Expression of PPARγ and C/EBPα ↓, Phosphorylation of AKT and ERK ↓ (b)	(Galvis et al., 2011) (a), (Abood et al., 2018) (b)
11,13-dihydro-[11S]-dehydroleucodine	3T3-L1	Inhibition	80 µM	9 days	-	Galvis et al. (2011)
(11*S*)- 3-oxoeudesmano-13,6α-lactone	HL-60	No response	100 µg/ml	3 days	-	Kim et al. (2006)
(11*S*)-3,3-(ethylenedioxy) eudesmano-13,6α-lactone	HL-60	No response	100 µg/ml	3 days	-	Kim et al. (2006)
Santonin	HL-60	No response	100 µg/ml	3 days	-	Kim et al. (2006)
Ainsliaside A	3T3-L1	Inhibition	10–40 µM	8 days	-	Kim et al. (2016)
Arsantin	HL-60	Induction	50 µg/ml	3 days	Phosphorylation p38, JNK, ERK ↑	Kweon et al. (2015)
Artesunate	3T3-L1 (a), human preadipocytes (b)	Inhibition (a), No response (b)	1–5 µM (a), 5 µM (b)	8 days (a), 20 days (b)	Expression of PPARγ and C/EBPα ↓ , Phosphorylation of STAT3 ↓ (a), - (b)	(Jang, 2016) (a, b)
Atractylenolide I	Murine melanoma B16 cells (a), Rat Mesenchymal stem cell (b), K562 CML (c), U937 AML (d)	Induction (a, b, c, d)	100 µM (a), 3–30 µg/ml (b), 6.25–50 µg/ml (c), 6.25–50 µg/ml (d)	3 days (a), 7 days (b), 3–6 days (c), 3–4 days (d)	Activation of ERK ↓ , Activation of p-AKT ↓ (a), Expression of shh and Gli-1 ↑ (b),–(c), - (d)	(Ye et al., 2011) (a), (Li et al., 2012) (b), (Huang et al., 2016) (c, d)
Atractylenolide II	Murine melanoma B16 cells	Induction	100 µM	3 days	Activation of ERK ↓ , Activation of p-AKT ↓	Ye et al. (2011)
Atractylenolide III	Rat mesenchymal stem cell	Induction	3–30 µg/ml	7 days	Expression SHH and GLI-1 ↑	Li et al. (2012)
Costunolide	BMM (a), CD4^+^ T cells (b)	Inhibition (a, b)	1–5 µM (a), 0.5–2 µM (b)	4 days (a), 2–3 days (b)	Expression of NFATc1↓ , Expression of c-Fos ↑(a), Activation of p38 and ERK ↓ (b)	(Cheon et al., 2014) (a), (Park et al., 2016)(b)
Cumambrin A	BMM	Inhibition	0.5–5 µM	5 days	Activation of NF-kB and NFATc1 ↓ , Activation of ERK ↓	Zhou et al. (2019)
Dehydrocostus lactone	BMM (a, b, c), K562 CML (d)	Inhibition (a, b, c), Induction (d)	0.5–2.5 µM (a), 1–4 µM (b), 0.5–1.5 µM (c), 2–8 µM (d)	5 days (a), 5 days (b), 4 days (c), 1 day (d)	Phosphorylation of IKKα/β, IκBα, p65 ↓, Activation and Expression of NFATc1 ↓ (a), Expression of NFATc1 ↓, Activation of IKK, p65, IκBα ↓ (b), Expression of NFATc1 ↓ (c), Expression of p-STAT3, p-STAT5, p-JAK2, p-BCR-Abl, BCR-Abl ↓ (d)	(Li et al., 2019) (a), (Hu et al., 2019) (b), (Lee et al., 2019) (c), (Cai et al., 2017) (d)
Dehydroleucodine	3T3-L1	Inhibition	4–10 µM	6–9 days	Expression of PPARγ and C/EBPα ↓, Phosphorylation of AMPKα ↑	Galvis et al. (2011)
Eupalinilide E	HSPCs	Inhibition	0.6 µM	7 days	-	de Lichtervelde et al. (2013)
Helenalin	HL-60	Induction	5–10 µM	3 days	Activation of PKC, ERK ↑ Expression of PKC h1 and h2 ↑, Activity of NF-κB ↓	Kim et al. (2005)
Isoalantolactone	BMM	Inhibition	0.5–2 µM	2–4 days	Expression of NFATc1, c-Fos ↓, Phosphorylation of JNK, p38, NF-κB, AKT ↓	Lu et al. (2020)
Parthenolide	Human periosteum-derived cells	No response	1–5 µM	10/21 day	-	Park et al. (2020)
Taraxinic Acid	HL-60	Induction	15–30 µM	4 days	Expression of c-myc ↓, Expression of p21CIP1, p27KIP1 ↑	Choi et al. (2002)
Zaluzanin C	3T3-L1 (a), Preosteoblast-MC3T3-E1 (b), Mesenchymal stem cells C3H10T1/2 (c)	Inhibition (a), Induction (b, c)	1–10 µM (a), 5 µM (b), 5 µM (c)	12 days (a), 2 days (b), 2 days (c)	Expression of PPARγ, FABP4 ↓ (a), Expression of Runx2, Id1, Dlx5 ↑ (b), Expression Runx2, Id1, Dlx5 ↑ (c)	(Kwak and Kim, 2019) (a), (Kim et al., 2017) (b, c)

BMM, bone marrow monocytes, CML, chronic myeloid leukemia, AML, acute myeloid leukemia.

### NFκB Pathway

NFκB is a family of transcription factors including five genes (NFκB1, NFκB2, RelA, c-Rel, and RelB) that encode seven proteins (p50, p105, p52, p100, p65, c-Rel, RelB). Many signaling pathways might activate the NFB pathway through classical or alternative pathways; either way, activated NFκB is translocated to the nucleus and acts as a transcription factor. Cell differentiation, cell survival, and inflammation may be affected by the NFκB pathway (Dolcet et al., 2005).

Included studies in this systematic review reported that sesquiterpene lactones also affect the NFκB pathway (Kim et al., 2005; Zhou et al., 2019; Li et al., 2019; Hu et al., 2019; Lu et al., 2020). The phosphorylation level of NFκB was decreased in response to sesquiterpene lactones with either inhibiting or inducing effects on differentiation (Table 1). Among five studies evaluating NFκB, one reported inducing effects (Kim et al., 2005), and the others reported inhibiting effects (Hu et al., 2019; Li et al., 2019; Zhou et al., 2019; Lu et al., 2020). In the downstream of NFκB, the expression level of NFATc1 has also been shown to be reduced by sesquiterpene lactones unanimously in all studies evaluating their effects (Cheon et al., 2014; Lee et al., 2019; Li et al., 2019; Zhou et al., 2019; Lu et al., 2020). Although the studies evaluating NFκB concluded that this pathway is responsible for the occurrence or blockage of differentiation (as in (Hu et al., 2019)), our observation on these data suggests that NFκB might be affected as a general response to sesquiterpene lactones, not directly related to differentiation. In other words, other pathways might be more effective, or the cumulative effects of a set of pathways might redirect the response. Moreover, the structural differences of sesquiterpene lactones, the physiological condition of cells, and the treatment condition in different studies are other parameters limiting the generalization. It is worth noting that two studies (Hu et al., 2019; Li et al., 2019) reporting inhibitory effects of dehydrocostus lactone on differentiation unanimously reported that dehydrocostus lactone does not affect the phosphorylation of NFκB.

### JAK-STAT Pathway

In mammals, JAK and STAT family consists of four (JAK1, JAK2, JAK3, TYK2) (Stark et al., 1998) and seven (STAT1, STAT2, STAT3, STAT4, STAT5a, STAT5b, STAT6) proteins, respectively (Copeland et al., 1995; Darnell, 1997). Different recruitment of JAKs and STATs is based on the cell types and receptors engaged in the signaling pathway (Rawlings et al., 2004). The binding of ligand to the JAK-binding transmembrane receptors allows them to multimerize, leading to JAK activation through trans-phosphorylation. Activated JAKs subsequently phosphorylate STATs. Phosphorylated STATs dimerize and translocate to the nucleus and act as transcription factors (Murray, 2007). Activated STATs have anti-apoptosis activity and also affect Cell-cycle progression, cell-cycle inhibition, Lipid metabolism, and differentiation according to KEGG database (Kanehisa and Goto, 2000; Kanehisa, 2019; Kanehisa et al., 2021). JAK-STAT pathway has been shown to be affected by sesquiterpene lactones in the studies included in this systematic review (Jang, 2016; Cai et al., 2017). The phosphorylation level of STAT3, STAT5, and JAK is reduced through both induction and inhibition of differentiation via sesquiterpene lactones (Table 1).

### PI3K-Akt Pathway

Phosphatydilinositol-3-kinase (PI3K) can be stimulated by many growth factors and regulators (Vivanco and Sawyers, 2002). Activated PI3K generates phosphatidylinositol-3,4,5-trisphosphate, which helps AKT to translocate to the plasma membrane where it gets phosphorylated and hence activated (Osaki et al., 2004; Hennessy et al., 2005). Activated PI3K/AKT pathway may play a pivotal role in cell cycle progression, apoptosis, and differentiation (Kanehisa, 2019; Kanehisa and Goto, 2000; Kanehisa et al., 2021; Delgoffe et al., 2011). Also, PI3K/AKT pathway can indirectly affect cellular differentiation by interfacing with other signaling pathways such as the NFκB pathway (through IKK activation) and JAK/STAT pathway (Kanehisa, 2019; Kanehisa and Goto, 2000; Kanehisa et al., 2021). The involvement of the PI3K-AKT pathway in response to three sesquiterpene lactones (i.e., atractylenolide I and II, 11,13-dihydro-[11R]-dehydroleucodine, isoalantolactone) through AKT has been reported (Ye et al., 2011; Abood et al., 2018; Lu et al., 2020). The level of phosphorylation of AKT was decreased in treatment with three lactones with either inhibiting or inducing effects (Table 1). However, one lactone (dehydroleucodine) failed to affect the phosphorylation of AKT (Galvis et al., 2011).

### PPARγ and C/EBPα Pathway

The expression of PPARγ and C/EBPα have been decreased in response to sesquiterpene lactones (Table 1). However, in one study, the expression of C/EBPα remained unchanged in response to zalzuzanin (Kwak and Kim, 2019). The involvement of the PPARγ pathway and C/EBPα was only reported in the studies investigating the inhibitory effects of sesquiterpene lactones. This might be because of the experimental designs as the PPARγ pathway and C/EBPα are involved in adipogenesis and lipid metabolism (Farmer, 2005; Lefterova et al., 2008), which were intended to be inhibited by sesquiterpene lactones. Therefore, still, the question remains that how the PPARγ pathway and C/EBPα are affected by sesquiterpene lactones in other cells, especially cancer cells.

### Miscellaneous Pathways

In one study, the expression of SHH and GLI was increased by atractrynolide with inducing effects on differentiation, indicating the possible involvement of Sonic-Hedgehog signaling pathway in the differentiation (Li et al., 2012). Sesquiterpene lactones are also found to decrease the expression of Fas involved in apoptosis (Jang, 2016; Abood et al., 2018), increase the phosphorylation and expression of PKC involved in signaling in general (Kim et al., 2005) and increase the expression of p27 and p21 and decrease the expression of c-Myc involved in the cell cycle (Choi et al., 2002; Abood et al., 2018).

### Biological Assays for Evaluating Differentiation

Physiological cellular differentiation is routed from comprehensive alteration of various hierarchical molecular mechanisms in the cells. Gene and protein profiles of the cells change during cellular differentiation or dedifferentiation, and miRNA profiles can also be indicative of differentiation status (Letzen et al., 2010; Branco et al., 2015; Frese et al., 2017). More primarily, chromatin remodeling is identified as a pivotal step in this process (Chen and Dent, 2014). Molecular-level changes would lead to functional and morphological changes, measurable by colorimetric and enzymatic assays, microscopy, and flow cytometry. The process of differentiation, as a continuous spectrum of events, as well as the final status of differentiation, can be measured at every molecular, functional, and morphological level (Clark, 1957; Chen et al., 1989; Moyes et al., 1997; Flaim et al., 2005). In this sense, evaluating various molecular, functional, and morphological levels of the cells is required for the most confident assessment of cellular differentiation.

Various types of assays such as morphological and molecular assays, microscopy, colorimetry, flow cytometry, and enzyme activity assay were applied in the selected studies to evaluate the status of differentiation. These assays were used for confirmation of differentiation or studying the underlying mechanism of it. Nineteen studies applied qRT-PCR or western blot techniques to assess the sesquiterpene lactones’ underlying mechanism of action. Only eight studies used flow cytometry to approve the status of differentiation. The assays used for evaluation of differentiation are summarized in the Table 2.

**TABLE 2:** Biological assay for evaluation of differentiation.

Treated Cell	Differentiated Cells	Biological assay	Markers
Inhibition of 3T3-L1 differentiation	-	Oil red O staining	Lipid droplet (Galvis et al., 2011; Jang, 2016; Kim et al., 2016; Abood et al., 2018; Kwak and Kim, 2019)
		Triglyceride assay	Triglyceride (Galvis et al., 2011; Jang, 2016; Abood et al., 2018)
		qRT-PCR	C/EBP-α (Jang, 2016), PPAR-γ (Jang, 2016), FAS (Jang, 2016), perilipin A (Jang, 2016), leptin (Jang, 2016), adiponectin (Jang, 2016)
		Western blot	C/EBP-α (Galvis et al., 2011; Jang, 2016; Abood et al., 2018; Kwak and Kim, 2019), PPAR-γ (Galvis et al., 2011; Jang, 2016; Abood et al., 2018; Kwak and Kim, 2019), STAT-3 (Jang, 2016), STAT-5 (Jang, 2016), FAS (Jang, 2016; Abood et al., 2018), perilipin A (Jang, 2016), AMPK (Galvis et al., 2011; Abood et al., 2018), Erk1/2 (Galvis et al., 2011), Akt 1 (Galvis et al., 2011), FABP4 (Kwak and Kim, 2019)
No effects on HL-60 differentiation	-	Nitro blue tetrazolium reduction assay	Superoxide generation (Kim et al., 2006)
		Morphology	Cell size and margin, morphological characteristics of nuclei (Kim et al., 2006)
		Cytofluorometric assay	CD11b, CD14 (Kim et al., 2006)
		Western blot	PI3k p85, PKC, ERK, NF-kB (Kim et al., 2006)
		PKC activity assay	PKC activity (Kim et al., 2006)
		Electrophoretic mobility shift assay	NF-kB (Kim et al., 2006)
		PKC activity assay	PKC activity (Kim et al., 2006)
No effects on human preadipocytes differentiation	-	Oil red O staining	Lipid droplet (Jang, 2016)
		Triglyceride assay	Triglyceride (Jang, 2016)
Inhibition of BMM differentiation	-	TRAP staining	(Cheon et al., 2014; Hu et al., 2019; Lee et al., 2019; Li et al., 2019; Zhou et al., 2019; Lu et al., 2020) (Hu et al., 2019)
		qRT-PCR	c-Fos (Cheon et al., 2014), NFATc1 (Cheon et al., 2014; Hu et al., 2019; Lee et al., 2019; Li et al., 2019; Lu et al., 2020), DAP12 (Cheon et al., 2014), FcRγ (Cheon et al., 2014), ACP5 (Cheon et al., 2014; Hu et al., 2019; Lee et al., 2019; Li et al., 2019; Zhou et al., 2019; Lu et al., 2020), OSCAR (Cheon et al., 2014; Hu et al., 2019), Ctsk (Hu et al., 2019; Zhou et al., 2019), CTR (Hu et al., 2019; Zhou et al., 2019), Cathepsin k (Hu et al., 2019; Li et al., 2019; Lu et al., 2020), CALCR (Lee et al., 2019; Li et al., 2019), DC-STAMP (Hu et al., 2019; Lee et al., 2019; Li et al., 2019; Lu et al., 2020), MMP9 (Hu et al., 2019; Lee et al., 2019; Lu et al., 2020), V-ATPASE a3 (Hu et al., 2019; Lu et al., 2020)
		Western blot	JNK (Cheon et al., 2014; Hu et al., 2019; Lu et al., 2020), p38 (Cheon et al., 2014; Hu et al., 2019; Lu et al., 2020), ERK (Cheon et al., 2014; Hu et al., 2019; Li et al., 2019; Zhou et al., 2019; Lu et al., 2020), IkB (Cheon et al., 2014; Hu et al., 2019; Li et al., 2019; Zhou et al., 2019; Lu et al., 2020), c-Fos (Cheon et al., 2014; Lu et al., 2020), NFATc1 (Cheon et al., 2014; Hu et al., 2019; Lee et al., 2019; Li et al., 2019; Lu et al., 2020), V-ATPASE-d2 (Li et al., 2019; Zhou et al., 2019), NF-kB (Li et al., 2019), p65 (Hu et al., 2019; Li et al., 2019; Lu et al., 2020), Cathepsin k (Hu et al., 2019; Lu et al., 2020), c-Src (Hu et al., 2019; Lu et al., 2020), AKT (Lu et al., 2020), TAK1 (Lu et al., 2020)
		luciferase gene assay	NF-kB (Zhou et al., 2019), NFAT (Li et al., 2019; Zhou et al., 2019)
Inhibition of CD4^+^ T cells differentiation	-	Flow cytometry	IFN-γ, IL4, IL13, IL17A (Park et al., 2016)
		ELISA	IFN-γ, IL4, IL13, IL17A (Park et al., 2016)
		qRT-PCR	IFN-γ, IL12R, IL4, IL13, IL17A, IL21, IL23R, T-bet, GATA3, RORγt (Park et al., 2016)
		Western blot	T-bet, GATA3, RORγt, ERK, p38 (Park et al., 2016)
Inhibition of HSPCs differentiation	-	Flow cytometry	CD71, glycophorin A (de Lichtervelde et al., 2013)
No effects on human periosteum-derived cells differentiation	-	ALP staining	ALP activity (Park et al., 2020)
		Alizarin red staining	Calcium content (Park et al., 2020)
Induction of preosteoblast-MC3T3-E1 differentiation	Osteoblast	qRT-PCR	Dlx5, Id1, Runx2 (Kim et al., 2017)
		Western blot	Runx2 (Kim et al., 2017)
		ALP staining	ALP activity (Kim et al., 2017)
		luciferase gene assay	Runx2 (Kim et al., 2017)
Induction of mesenchymal stem cells C3H10T1/2 differentiation	Osteoblast	qRT-PCR	Dlx5, Id1, Runx2 (Kim et al., 2017)
		Western blot	Runx2 (Kim et al., 2017)
		ALP staining	ALP activity (Kim et al., 2017)
		luciferase gene assay	Runx2 (Kim et al., 2017)
Induction of HL-60 differentiation	granulocyte (Kim et al., 2005; Kweon et al., 2015), Monocyte/macrophage (Choi et al., 2002)	Nitro blue tetrazolium reduction assay	Superoxide generation (Choi et al., 2002; Kim et al., 2005; Kweon et al., 2015)
		Phagocytosis test	Phagocytosis (Choi et al., 2002)
		Esterase activity test	Esterase activity (Choi et al., 2002)
		Morphology	Cell size and margin, morphological characteristics of nuclei (Kim et al., 2005; Kweon et al., 2015)
		Cytofluorometric assay	CD11b and CD14 (Kim et al., 2005; Kweon et al., 2015)
		Flow cytometry	CD14 and CD 66b (Choi et al., 2002)
		Western blot	PKC and ERK (Kim et al., 2005; Kweon et al., 2015), JNK and p38 MAPK (Kweon et al., 2015)
		Immune blot	c-myc, p27^KIP1^, p21^CIP1^ (Choi et al., 2002)
		Electrophoretic mobility shift assay	NF-kB (Kim et al., 2005)
		PKC activity assay	PKC activity (Kim et al., 2005)
Induction of murine melanoma B16 cells differentiation	B16 cells with dendrite like protrusions and increased tyrosinase activity and melanin content	Morphology	Dendrite like cellular protrusions (Ye et al., 2011)
		Tyrosinase activity assay	Tyrosinase activity (Ye et al., 2011)
		Measurement of cellular melanin	Cellular melanin content (Ye et al., 2011)
		Western blot	ERK, AKT (Ye et al., 2011)
Induction of rat Mesenchymal stem cell differentiation	Chondrocyte	qRT-PCR	Collagen II, aggrecan, SHH, Gli-1 (Li et al., 2012)
		Western blot	Collagen II, aggrecan, SHH, Gli-1 (Li et al., 2012)
		Immunohistochemical techniques	Collagen II, aggrecan (Li et al., 2012)
Induction of K562 CML differentiation	Monocyte/macrophage	Morphology	Shape of cell and nuclei (Cai et al., 2017)
		Flow cytometry	CD68 (Huang et al., 2016), CD14 (Huang et al., 2016; Cai et al., 2017), CD11b (Cai et al., 2017)
		Phagocytosis test by flow cytometry	Phagocytosis (Huang et al., 2016)
		Western blot	BCR-ABL, STAT-3, STAT-5, JAK2 (Cai et al., 2017)
Induction ofU937 AML differentiation	Monocyte/macrophage	Flow cytometry	CD68 and CD14 (Huang et al., 2016)
		Phagocytosis test by flow cytometry	Phagocytosis (Huang et al., 2016)

BMM, bone marrow macrophage, CML, chronic myelocytic leukemia, AML, acute myelocytic leukemia.

In some cases, the assays were not sufficient to confirm the differentiation status with high certainty. Differentiation and dedifferentiation affect all aspects of cellular physiology initiated from the upstream. Evaluating limited aspects of the cells would not necessarily demonstrate the status of differentiation. As an example, limited morphological studies or alteration in specific markers do not prove the redirection of cellular fate into a new differentiation status (Alt et al., 2011). Flow cytometry, gene expression (e.g., RT-PCR, and RNA-Seq), protein expression (e.g., immunoblotting, enzyme-linked immunosorbent assay, and mass spectrometry), imaging (e.g., electron microscopy, immunocytochemistry), specific staining techniques, cellular function assay, and motility assay can be used to confirm the differentiation status more accurately. Using these techniques together may further increase the accuracy. It is also worth noting that all the studies were biased to use the assays to confirm their primary hypothesis and not to rule out the other possibilities (Pannucci and Wilkins, 2010). The assays, although insufficient, demonstrated that the cells were differentiated or dedifferentiated; however, some questions still are remained: What are the other effects of sesquiterpene lactones on cellular physiology (e.g., metabolism, gene expression, and proliferation)? Will all these other effects be in line with the observed status of differentiation? How does one sure the treated cells do not obtain other characteristics during treatments rather than those assumed primarily?

### Overlooking Negative Results and Potential Misconception in Systematic Reviews

Both induction and inhibition of differentiation are considered therapeutic approaches in specific conditions. Not surprisingly, the results of all studies were in line with their objectives, either inhibiting or inducing effects. Only some studies (Kim et al., 2006; Jang, 2016; Park et al., 2020) reported the negative results, which were either evaluating and comparing the effects of other sesquiterpene lactones or non-lactone derivatives, or the primary objective of the study was not evaluating the differentiation. Moreover, the occurrence of reverse effects was not reported in any studies. The observation above might relate the differentiation status to the cells’ condition (i.e., cancerous or normal), leading to a potential misapprehension that sesquiterpene lactones induce differentiation in normal cells and inhibit it in cancer cells. However, this seems unlikely and biased since, in contrast to clinical researches, there is a much less tendency to report negative results, and many journals do not consider them in this field.

It has been a long time that researchers have expressed their concerns regarding the under-reporting of negative results which generally arises form “publication bias” (Earp, 2018). In fact, data from a 2012 study of more than 4,000 published papers show that scientific literature as a whole is trending towards more reports of positive results reached to more than 85% of published studies by 2007 (Mehta, 2019). In this case, a lucky lab with a positive result among many other labs with negative results have a chance to publish their findings, leading to a misconception; however, a cumulative evaluation of both positive and negative results could have unveiled that the positive result was a fluke, a basic statistical issue (Earp, 2018). In the absence of negative results, obtaining true-positive conclusions through systematic reviews and meta-analysis would be devastated. Interestingly, a simulation study confirmed that a higher probability of including positive results would increase the magnitude of the effect of publication bias, leading to an increase of the false-positive rate in meta-analysis (Kicinski, 2014).

The current approach for *in vitro* studies would slacken the progress in producing knowledge about sesquiterpene lactones’ effects on various cells and their underlying mechanism of action. Therefore, the answer to why sesquiterpene lactones affect differentiation differently remains unclear. Moreover, as a result, the translation of these molecules to *in vivo* studies and human trials will be limited. Waste of resources on not-reported failed experiments and manipulation of data to get papers published would be other problems rise from under-reporting negative results (Mehta, 2019).

## Conclusion

Sesquiterpene lactones can both inhibit and induce differentiation in various cells. Their differentiating/dedifferentiating effects are mediated through JAK-STAT, PI3K-Akt, MAPK, NFκB, PPARγ pathways. Moreover, they interfere with the cell cycle and apoptosis. However, extensively biased study design, under-reporting negative results (publication bias), a significant gap of knowledge regarding the other types of cells, sesquiterpene lactones, and involved pathways hamper a robust conclusion on the effects of sesquiterpene lactones and their mechanism of actions. Future studies should address these issues to prepare the knowledge basis and facilitate the translation of sesquiterpene lactones to the clinic.

## Data Availability

The raw data supporting the conclusion of this article will be made available by the authors, without undue reservation.
