# Supplementary material for: The Effects of Sesquiterpene Lactones on the Differentiation of Human or Animal Cells Cultured In-Vitro: A Critical Systematic Review
Source: Front Pharmacol. 2022 Apr 4;13:862446. doi: 10.3389/fphar.2022.862446 (PMC9014292; doi:10.3389/fphar.2022.862446)
Supplement: Supplementary file 1 [file Table1.DOCX]

# Search Strings:

# Scopus:

# TITLE-ABS-KEY ( ( sesquiterpen OR sesquiterpenes OR sesquiterpene OR sesquiterpenic OR sesquiterpens ) AND ( lacton OR lactones OR lactone OR lactonic OR lactonization OR lactonizations OR lactonize OR lactonized ) AND ( "cell differentiation" OR "cellular differentiation" OR "differentiated cell" OR differentiate OR differentiated OR differentiation OR differentiations OR differential OR differentials OR differentiating OR differentiates OR differentiational OR differentiative ) ) AND ( LIMIT-TO ( LANGUAGE , "English" ) )

# MEDLINE:

# (("sesquiterpen"[All Fields] OR "sesquiterpenes"[MeSH Terms] OR "sesquiterpenes"[All Fields] OR "sesquiterpene"[All Fields] OR "sesquiterpenic"[All Fields] OR "sesquiterpens"[All Fields]) AND ("lacton"[All Fields] OR "lactones"[MeSH Terms] OR "lactones"[All Fields] OR "lactone"[All Fields] OR "lactonic"[All Fields] OR "lactonization"[All Fields] OR "lactonizations"[All Fields] OR "lactonize"[All Fields] OR "lactonized"[All Fields]) AND ("cell differentiation"[MeSH Terms] OR (("cell"[All Fields] OR "cellular"[All Fields]) AND "differentiation"[All Fields]) OR "cell differentiation"[All Fields] OR "cellular differentiation"[All Fields] OR "differentiated"[All Fields] OR "differentiation"[All Fields] OR "differential"[All Fields] OR "differentials"[All Fields] OR "differentiate"[All Fields] OR "differentiates"[All Fields] OR "differentiating"[All Fields] OR "differentiational"[All Fields] OR "differentiations"[All Fields] OR "differentiative"[All Fields])) AND (english[Filter])

# Web of Science:

TOPIC: (((((((sesquiterpene OR sesquiterpenes) OR sesquiterpene) OR sesquiterpenic) OR sesquiterpene) AND (((((((lacton OR lactones) OR lactone) OR lactonic) OR lactonization) OR lactonization) OR lactonized) OR lactonized)) AND (((((((((((("cell differentiation" OR "cellular differentiation") OR "differentiated cell") OR differentiate) OR differentiated) OR differentiation) OR differentiations) OR differential) OR differentials) OR differentiating) OR differentiates) OR differentiation) OR differentiative)))

Refined by: LANGUAGES: ( ENGLISH )
